# Intraspecific convergence of floral size correlates with pollinator size on different mountains: a case study of a bumblebee-pollinated *Lamium* (Lamiaceae) flowers in Japan

**DOI:** 10.1186/s12862-021-01796-8

**Published:** 2021-04-24

**Authors:** Tsubasa Toji, Natsumi Ishimoto, Shin Egawa, Yuta Nakase, Mitsuru Hattori, Takao Itino

**Affiliations:** 1 Graduate School of Medicine, Science and Technology, Shinshu University, Matsumoto, 3-1-1 Asahi, Nagano, 390-8621 Japan; 2 Faculty of Science, Shinshu University, Matsumoto, 3-1-1 Asahi, Nagano, 390-8621 Japan; 3 Graduate School of Fisheries and Environmental Sciences, Nagasaki University, 1-14 Bunkyo-machi, Nagasaki, 852-8521 Japan; 4 Department of Biology and Institute of Mountain Science, Shinshu University, Matsumoto, 3-1-1 Asahi, Nagano, 390-8621 Japan

**Keywords:** Bumblebee, Flower size, Independent evolution, Pollination, Trait matching

## Abstract

**Background:**

Geographic differences in floral size sometimes reflect geographic differences in pollinator size. However, we know little about whether this floral size specialization to the regional pollinator size occurred independently at many places or occurred once and then spread across the distribution range of the plant species.

**Results:**

We investigated the relationship between the local floral size of flowers and local pollinator size in 12 populations of *Lamium album* var. *barbatum* on two different mountains in the Japan Alps. Then, using 10 microsatellite markers, we analyzed genetic differentiation among the 12 populations. The results showed that local floral size was correlated with the average size of relevant morphological traits of the local pollinators: floral size was greater in populations visited frequently by the largest flower visitors, *Bombus consobrinus* queens, than it was in other populations. We also found that the degree of genetic similarity between populations more closely reflected interpopulation geographic proximity than interpopulation similarity in floral size.

**Conclusions:**

Although genetic similarity of populations was highly associated with geographic proximity, floral size varied independently of geographic proximity and was associated with local pollinator size. These results suggest that in *L. album* var. *barbatum*, large floral size evolved independently in populations on different mountains as a convergent adaptation to locally abundant large bumblebee species.

**Supplementary Information:**

The online version contains supplementary material available at 10.1186/s12862-021-01796-8.

## Background

Plant–pollinator interaction, one of the main mutualistic relationships between angiosperms and animals, greatly influences the reproductive success of plants [1–7]. Floral adaptation to pollinators is thought to be a key mechanism leading to the diversification of flower traits and speciation in angiosperms [8–11]. Accordingly, variations in floral characteristics, including in flower shape [12, 13], size [14, 15], color [16, 17], and odor [18, 19], have been recognized to have resulted from adaptation to pollinators. In fact, many studies have shown that geographic variation of flower traits is associated with geographic variation of pollinator assemblages [4, 13, 20–32]. These have been interpreted as the consequences of adaptation of floral traits to pollinators.

Local adaptation of plants to pollinators can lead to plant speciation through the establishment of prezygotic reproductive isolation, because specialization to specific pollinators may preclude pollinator sharing between related plant lineages [4, 22, 33]. In fact, according to the Grant–Stebbins model of floral divergence [8, 9, 11, 34], prezygotic reproductive isolation through pollinator-based selection is the main pathway of floral trait diversification. The Grant–Stebbins model proposes that local adaptation of plants to local pollinator assemblages results in trait diversification and reinforcement of reproductive isolation. Thus, a geographic mosaic of flower visitors may promote allopatric divergence of plants leading to the emergence of different ecotypes. Accordingly, if divergence in allopatry is followed by secondary contact, we can hypothesize that local adaptation to pollinators may prevent gene flow between the two ecotypes even after the secondary contact [18, 19]. One useful approach to understanding trait diversification and speciation in angiosperms, therefore, is to combine an ecological evolutionary analysis of local plant adaptations with an analysis of population genetics to assess the degree of genetic isolation between populations. Given that about 25% of angiosperm diversification events may be associated with a shift in pollinators [35], this combination of analytical approaches can shed considerable light on the origin of plant diversity [36, 37]. Nevertheless, researchers focusing on plant diversification have only recently begun to use these two approaches in combination [34, 38, 39]. In particular, knowledge of the patterns of morphological changes associated with intraspecific genetic structures can contribute to our understanding of the early stages of divergence [34].

In this study, we posit two hypotheses to explain geographic differences in floral characteristics. The first hypothesis is ‘secondary contact’ hypothesis. It assumes that allopatric floral size differentiation occurred between populations with large-sized flowers where plants were pollinated by large pollinators, and populations with small-sized flowers where plants were pollinated by small pollinators. In this scenario, the different-sized flowers have already been reproductively isolated because of the different pollinators, their distribution range secondarily overlapped, and currently gene flow occurs only between similar-sized flowers. The second hypothesis is ‘independent local adaptation of floral size’. In contrast to ‘secondary contact’ hypothesis, it assumes that the local floral size is the results of current adaptation selected by local pollinator size and the gene flow occurs mainly between nearby populations because no reproductive isolation between different-sized flowers evolved yet. In this scenario, the degree of genetic similarity among populations should reflect geographic proximity rather than floral size similarity. Based on this hypothesis, we assume that the floral size has evolved independently among mountain regions.

*Lamium album* (Lamiaceae) is native to Europe and Asia. In Europe, it is reported to be visited mainly by bumblebees, small wild bees and honeybees [40]. The Asian subspecies, *L. album* var. *barbatum*, is visited mainly by bumblebees [41]. In Japan, floral size varies geographically in *Lamium album* var. *barbatum* [41]. Flower–pollinator trait matching has been demonstrated in a Japanese population of *L. album* var. *barbatum* by Hattori et al. [42], who observed that as the difference between bumblebee tongue length and the floral size of *L. album* var. *barbatum* becomes larger in a population, fruit set per single pollinator visit becomes smaller. Thus, we expect floral size to be greater in Japanese populations of *L. album* var. *barbatum* visited by larger pollinators, and we can expect to find a relationship between floral size and the size of relevant pollinator traits in those populations.

In this study, we investigated the relationship between floral size and pollinator size in 12 populations of *L. album* var. *barbatum* in two different mountain areas and confirmed plant–pollinator trait matching in these populations: plants in populations visited by long-tongued pollinators characteristically had long corolla tubes, whereas plants in populations visited by short-tongued pollinators had short corolla tubes. In addition, using 10 microsatellite markers, we estimated the population genetic structures of the 12 *L. album* var. *barbatum* populations and found that floral size correlated with local pollinator size but not with the genetic similarity of populations. This finding supports convergent intraspecific floral trait evolution: the second of the two hypotheses formulated above.

## Results

### Geographic variation of floral size

We found that floral size of *L. album* var. *barbatum* and the pollinator assemblage greatly differed among populations (Tukey's HSD, *P* < 0.05; Table 1). There was no spatial autocorrelation of average floral size between populations (Moran's I = –0.028; *P* = 0.332).

**Table 1:** Survey results from the 12 *L. album* var. *barbatum* populations

	Location											
	West area							East area				
	Shimashima I	Shimashima II	Ohmizusawa	Onosawa	Mitsumata	Norikura	Ougisawa	Fujiidani	Santanda	Ushifuse	Nakayamasawa	Hirokoba
Visitation frequency												
Small bees (whole-body pollinators) total	7	87	189	20	7	13	–	29	26	28	16	–
Large bees (thrust pollinators) total	0	7	63	3	28	61	4	33	2	16	33	2
*Eucera* ssp. & *Apis* ssp.	–	7	–	–	–	–	–	33	2	16	–	–
*Bombus ardens* worker	–	–	–	–	–	12	–	–	–	–	–	–
*B. honshuensis* worker	–	–	14	–	–	40	–	–	–	–	4	–
*B. honshuensis* queen	–	–	29	–	1	–	–	–	–	–	4	–
*B. diversus* worker	–	–	–	3	–	1	–	–	–	–	12	–
*B. diversus* queen	–	–	–	–	–	–	–	–	–	–	1	–
*B. consobrinus* worker	–	–	3	–	–	8	2	–	–	–	12	1
*B. consobrinus* queen	–	–	17	–	27	–	2	–	–	–	–	1
Observation time (min)	540	230	600	270	215	357	90	450	450	410	660	130
Visitation rate (individual/h)	0.78	26.35	31.50	5.78	17.58	22.69	5.33	12.67	4.00	8.78	7.45	1.85
Average pollinator size (mm, all visitors)	11.38	11.78	13.41	10.05	23.61	14.05	24.91	12.44	11.91	12.57	15.72	23.46
Average pollinator size (mm, only large bees)	–	13.45	19.11	17.48	26.72	15.43	24.91	12.66	12.08	13.12	17.73	23.46
Average pollinator size (mm, only small bees)	11.38	11.65	11.60	11.11	11.18	8.85	–	12.20	11.89	12.26	11.59	–
Average floral size (mm ± SD)	25.91 (± 0.57)^a^	28.57 (± 0.90)^d^	29.21 (± 1.44)^e^	27.71 (± 0.76)^c^	30.53 (± 1.26)^fg^	28.55 (± 1.03)^d^	31.12 (± 0.92)^g^	27.06 (± 0.86)^b^	25.93 (± 0.79)^a^	26.78 (± 0.84)^b^	28.32 (± 1.35)^d^	30.01 (± 1.18)^f^
Census days	4 Apr–16 May 2018	20 May–6 Jun 2018	11 May –18 Jun 2018	1–6 Jun 2019	4–14 Jun 2019	17 Jun–9 Jul 2018	26 Jun–3 Jul 2019	17–23 May 2019	2–30 May 2019	10–31 May 2018	1–12 Jun 2019	21 Jun–1 Jul 2019

Pollinator visitation frequencies in each 1 m × 1 m quadrat during the indicated observation time. The census days of each population are within the approximate peak flowering period of that population. Different lowercase letter superscripts to average floral size indicate significant differences between the populations (Tukey's honestly significant difference (HSD) test, *P* < 0.05)

### Pollinator size variation

In the survey of insect visitors, large bees, small bees (whole-body pollinators), small bees (without attached pollen grains), and nectar robbers were observed (Additional file 1: Table S1). In particular, only small bees visited flowers of the Shimashima I population. In contrast, only large bees visited flowers of the Ougisawa and Hirokoba populations. In our analysis, we treated only the first two groups as valid pollinators. The average pollinator size varied among populations: for all pollinators (first two groups only), it was 10.05–24.91 mm; for large bees, it was 12.08–26.72 mm, and for small bees, it was 8.85–12.26 mm (Table 1). The largest bees were queens of *Bombus consobrinus*, which were observed in particularly high proportions in the Mitsumata, Ougisawa, and Hirokoba populations (Additional file 1: Table S1). Bees that were not considered to contribute to pollination were excluded from the size measurements. These included small bees without attached pollen grains (*E. nipanicus*, *L. nipponense*, *L. occidens*, *N. comparata*, and *Nomada* spp.), which were observed only at Onosawa and Fujiidani, and nectar robbers (*A. mellifera*, *B. hypocrita*, *X. appendiculata circumvolans*), which forage for nectar by drilling a hole in the lower part of the corolla tube (Additional file 1: Table S1).

### Factors influencing local floral size

**Fig. 1:**
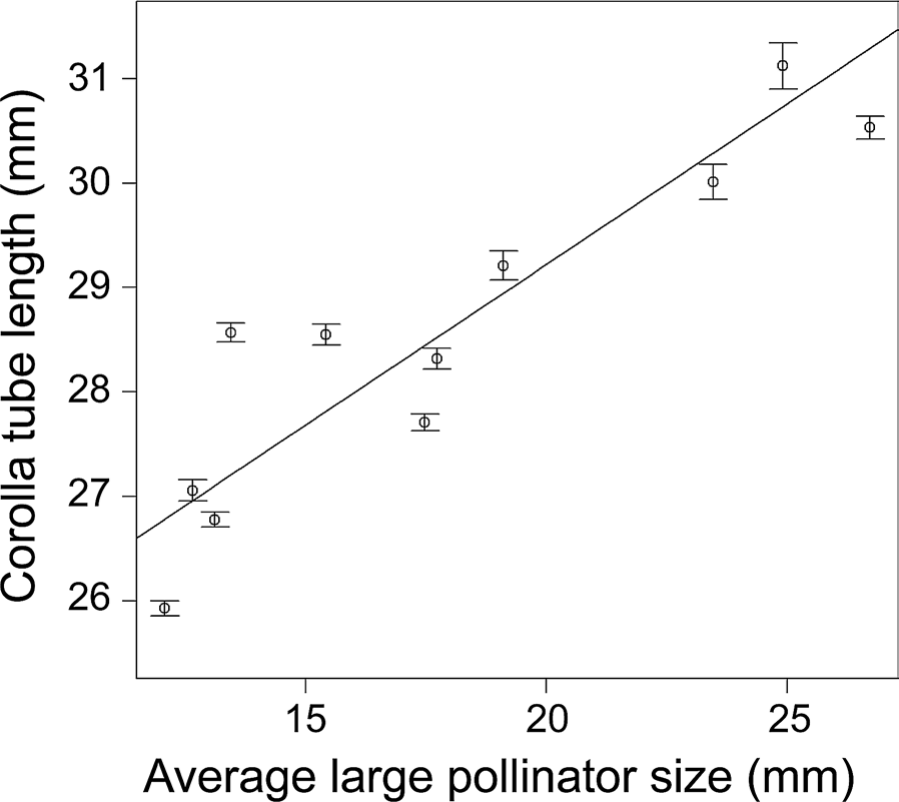
Relationship between floral size and average size of large bee pollinators. The line was fitted to the data by LMM result (*P* < 0.001). Data for Shimashima I, where no large bees visited the flowers, were not included in the regression analysis and are not shown in the figure. Error bars for average population floral size indicate the standard error

As a variable selection result, average pollinator size (only small bees) and plant height were selected as ineffective variables, so these variables were excluded from LMM analysis (Likelihood ratio test, *P* < 0.01). The model with the lowest Akaike information criterion (AIC) value and occupied high weight was that in which the average pollinator size (only large bees) was included as only predictive variable (Additional file 2: Table S2). In this model, the average pollinator size (only large bees) was a statistically significant variable (Table 2). By a regression analysis between floral size and the average pollinator size (only large bees), we detected a strong relationship (least squares regression, *R*^2^ = 0.807, LMM, *P* < 0.001; Fig. 1).

**Table 2:** Outcome of the linear mixed model with the lowest AIC value (Additional file 2: Table S2)

Factor	Coefficient	SE	*t*	*P*-value
Intercept	23.07	0.532	43.39	0.009
average pollinator size (only large bees) (mm)	3.076 × 10^–1^	8.031 × 10^–3^	38.31	2.00 × 10^–16^

Testing the effect of the average pollinator size (only large bees) to floral size of *L. album* var. *barbatum*

### Genetic structure of *Lamium album* var. *barbatum* populations

**Fig. 2:**
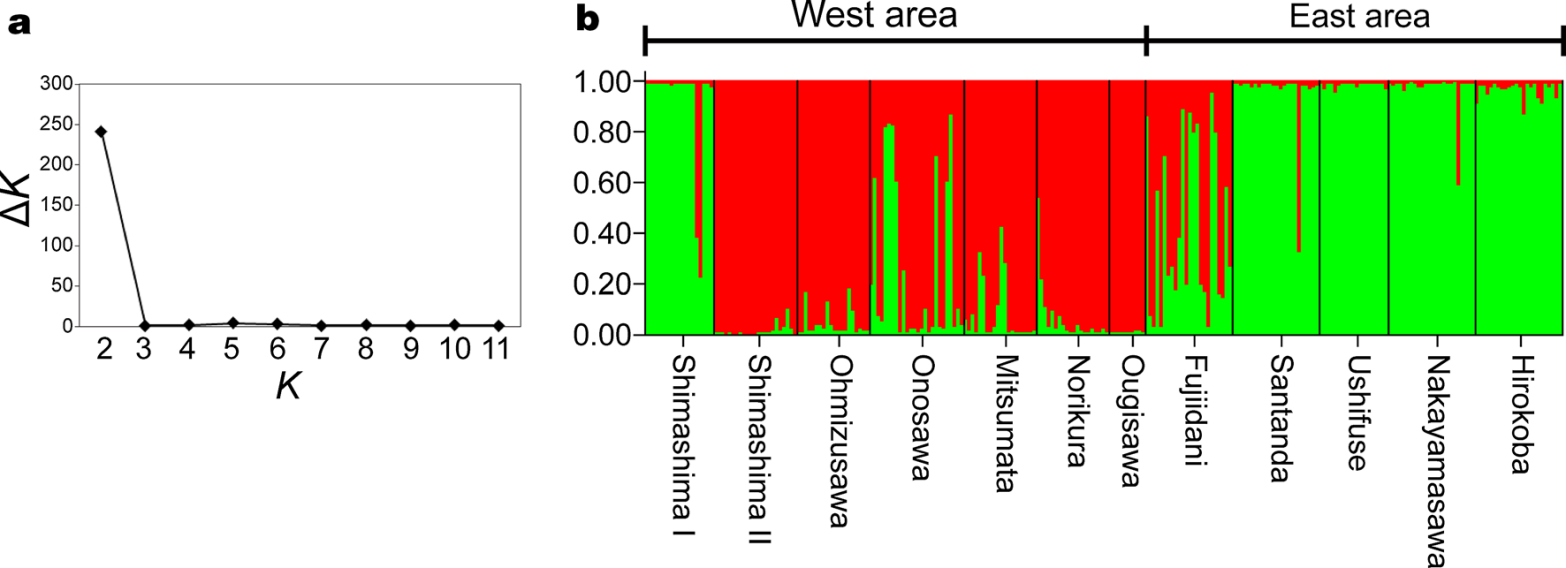
Population genetic structure of *L. album* var. *barbatum* populations. **a** Δ*K*, an index used to determine the appropriate number of genetic clusters (*K*), peaked at *K* = 2. **b** Genetic structure of *L. album* var. *barbatum* inferred by using Bayesian clustering implemented in STRUCTURE with *K* = 2. Different genetic clusters are represented by different colors

The analysis of molecular variance (AMOVA) result based on 10 microsatellite loci also indicated a significant difference in genetic structure between the two mountain areas (Table 3; *Φ*_CT_ = 0.031; *P* < 0.022). However, in the AMOVA result, most of the genetic variation was detected within populations (79.56%) and among populations within areas (17.31%). In the STRUCTURE analysis result, the most appropriate number of genetic clusters was *K* = 2 (Fig. 2a), and, for the most part, the populations in the east area were found to differ genetically from those in the west area (Fig. 2b). However, the Shimashima I population, although located in the west area, was genetically closer to populations in the east area, whereas the Fujiidani population, which was in the east area, was genetically closer to populations in the west area.

**Table 3:** Analysis of molecular variance (AMOVA) results for the 12 *L. album var. barbatum* populations

Source of variance	df	SS	Variation (%)	*Φ* statistic	*p*-value
Among mountain areas: west and east area	1	34.52	3.13	*Φ*_CT_ = 0.031	0.022
Among populations within areas	10	170.24	17.31	*Φ*_SC_ = 0.179	< 0.001
Within populations	494	832.75	79.56	*Φ*_ST_ = 0.204	< 0.001
Among floral size groups: based on Tukey's HSD	5	92.231	− 1.10	*Φ*_CT_ = -0.001	0.621
Among populations within floral size groups	6	112.528	20.28	*Φ*_SC_ = 0.201	< 0.001
Within populations	494	832.749	80.81	*Φ*_ST_ = 0.192	< 0.001

## Discussion

### Relationship between floral size and pollinator size

**Fig. 3:**
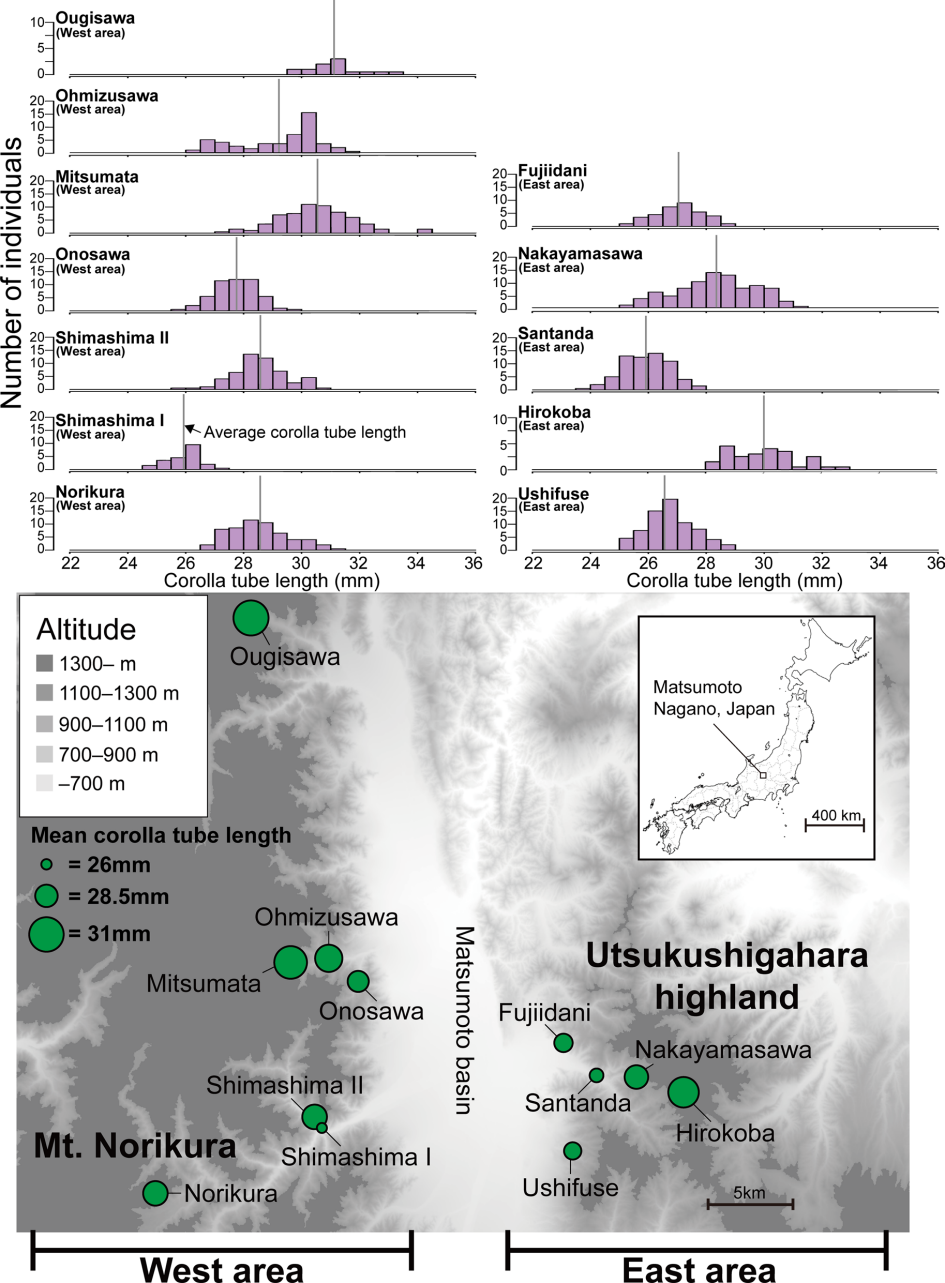
Study sites and mean floral size in each population. Distribution of floral size in the 12 populations (top) and the locations of the studied *L. album* var. *barbatum* populations (bottom). The vertical gray line in each histogram indicates the average floral size in that population. The size of the circles on the map indicates the average floral size of the indicated population. The west area comprises populations in the Mt. Norikura region, and the east area comprises populations in the Utsukushigahara highland region. This map is based on the Digital Topographic Map published by Geospatial Information Authority of Japan (https://www.gsi.go.jp/)

Both the floral size and pollinator assemblages of *L. album* var. *barbatum* showed geographic variations (Table 1; Fig. 3), but the lack of any spatial autocorrelation of floral size suggests that populations that are spatially close are not necessarily similar in floral size. In fact, the model that best explained floral size of a population was that in which the average size of large bees was the only explanatory variable (Table 2). Moreover, in the regression analysis of the 12 populations, floral size was strongly correlated with the average size of large bee pollinators (Fig. 1).

Unlike large bees, small bees can forage successfully in flowers with both short and long corolla tubes because they crawl into the flower tube to forage. Therefore, a match between the body size of small bees and floral size is not necessary for successful pollination. Interspecific variation in body size and tongue length is a prominent feature of large bees, *Bombus* spp., and many studies have demonstrated correlations between floral size in a plant species and the *Bombus* species composition of its pollinator assemblage [3, 13, 31, 43]. Our results indicate that in *L. album* var. *barbatum*, floral size at a particular location is correlated with the local average body size of large bees. However, it is possible that the correlation between floral size and local pollinator size reflects selection on a co-varying characteristic or selection mediated by other agents [44]. In this context, the observation that the correlation between floral size and local pollinator size was associated with seed set per single visit by a bumblebee in a *L. album* var. *barbatum* population at Norikura [42] is good evidence that variation in this floral trait represents an adaptation to pollinator size.

At the Mitsumata and Hirokoba locations, the herb *Meehania urticifolia*, which has a long corolla tube (over 40 mm), was abundant, and *B. consobrinus* queens visited the flowers of this herb during its flowering season, just prior to that of *L. album* var. *barbatum*. Similarly, at Ougisawa, the shrub *Weigela hortensis*, which also has a long corolla tube, blooms a little earlier than *L. album* var. *barbatum*, and *B. consobrinus* queens were observed to visit flowers of both species (T. Toji personal observation). Thus, at sites with populations of *L. album* var. *barbatum* flowers having long corolla tubes, other flower species also tended to have long corolla tubes. These observations suggest that the local evolution of long floral size in *L. album* var. *barbatum* may reflect interactions with large bumblebees in these local areas.

Our results add to this classic flower-pollinator trait matching result [4, 13, 20–32], and we show that the selection by flower visitors is the evolutionary background of change in floral size (Fig. 1). The mechanism through which pollinators exert the selective pressures have been shown to be selection in pollen export. A recent meta-analysis has shown that the amount of investment in petals evolves via strong competition for pollen export [45]. It is conceivable that this complex of factors may have resulted in selection for floral size. However, the very strong linear relationship between floral size and pollinator size still suggests that selection by flower visitors is the evolutionary background of floral size (Fig. 1).

### Genetic structure and independent floral size adaptation

The STRUCTURE analysis and AMOVA results suggest that, in general, populations within each mountain area were more closely related to each other than they were to populations in the other mountain area (Table 3; Fig. 2; Additional file 3: Figure S1). The largest flower visitors, *B. consobrinus* queens, visited four populations, Ohmizusawa, Mitsumata and Ougisawa in the west area and Hirokoba in the east area, and floral size in these four populations was significantly longer than it was in other populations (Table 1). However, in the genetic clustering analysis results, Ohmizusawa, Mitsumata and Ougisawa belonged to one of the two genetic clusters detected whereas Hirokoba belonged to the other (Fig. 2). This result suggests that floral size in *L. album* var. *barbatum* evolved independently in each genetic cluster.

The large genetic gap between the Shimashima I and Shimashima II populations is interesting because these two populations are only 0.4 km apart in straight line distance (Fig. 3). This genetic difference may reflect a history of colonization. In these two populations, *L. album* var. *barbatum* plants bloom at different times of the year (Table 1), and the pollinator assemblages and floral size distributions also differ between them. Given these differences in the timing of flowering and in the flower visitor assemblages, we infer that these populations are able to maintain genetic independence despite their proximity. Similarly, in Matsumoto, Japan, the shrub *Cimicifuga simplex* comprises multiple parapatric ecotypes that appear to be maintained by differences in the flowering season and flower visitor assemblage among the ecotypes [18, 46]. Further study is needed to determine what factors maintain the genetic differentiation between the Shimashima I and II populations in *L. album* var. *barbatum*. Although Shimashima I is located in the west area, it is genetically more closely related to populations in the east area. Similarly, Fujiidani is in the east area but is genetically more closely related to populations in the west area (Fig. 2b). Clear evidence to explain these discrepancies in the genetic structure of these populations is currently lacking.

The most striking aspect of our results is that the evolutionary geographic mosaic displayed by flower tube length variation reflects the regional distribution of the large bumblebee *B. consobrinus*, whereas the genetic similarity among populations reflects geographic proximity rather than flower trait similarity. Our results thus support the second hypothesis (floral size ecotypic ‘speciation’ did not occur, and trait divergence is independent of population genetic structure: convergent intraspecific floral trait evolution) proposed in the introduction. Sympatric ecotypic divergence in different mountain areas in Japan has also been reported in the alpine herb *Potentilla matsumurae* [47]. In this species, two ecotypes have been found, one favoring growth in fellfields and the other favoring growth in snowbeds. This ecotype divergence has occurred independently in at least two geographically separated mountain areas in Japan (Hokkaido and Tohoku), and the different ecotypes in the same region are genetically close. This pattern is similar to the results of this study. Thus, the independent divergence of floral traits can be detected by comparing floral traits and genetic structures across mountain ranges.

## Conclusions

We presented evidence for convergent intraspecific floral trait evolution by showing that changes in floral morphology in populations of *L. album* var. *barbatum* were associated with a shift to a morphologically different pollinator assemblage, but did not reflect the degree of genetic relatedness among the *L. album* var. *barbatum* populations. This study showed that a comparative approach to plant traits and genetic structure between mountain areas can be useful for demonstrating intraspecific genetic divergence and convergence of plant traits. To verify the Grant-Stebbins model, described in the introduction, it will be necessary in the future to examine a larger clade with more transitions in pollinating systems together with information on pollinator ranges, plant migration patterns (biogeography), and the direction of pollination system transitions [39].

## Methods

### Plant species

*Lamium album* L. var. *barbatum* (Lamiaceae) is a perennial herb that grows along forest edges throughout East Asia [48]. It produces creamy white, two-lipped, entomophilous, and self-incompatible flowers [40, 41]. The flowers are frequently visited by various bumblebee species, and in Japan, bumblebees are their main pollinators [41]. Flower–pollinator morphological matching has been reported to improve seed set in a population of *L. album* var. *barbatum* located near the populations of this study [42]. A bumblebee visiting a flower of *L. album* var. *barbatum* inserts its tongue into the inner part of the corolla tube to forage for nectar and in the process rubs its head and thorax against the anthers and the stigma. In addition to bumblebees, honeybees and wild bees have been observed to visit European (Poland) *L. album* flowers [40].

### Study site

Populations of *L. album* var. *barbatum* were surveyed at 12 sites in two mountain areas in Matusmoto, Nagano Prefecture, the central Japan Alps. All surveys were conducted between April and July, during the flowering season of each population, in 2018 or 2019. The two mountain areas were around Mt. Norikura, west of the Matsumoto basin (the "west area"), and around the Utsukushigahara highland, which is east of the basin (the "east area") (Fig. 3). Each population of *L. album* var. *barbatum* was a geographically cohesive group of densely distributed plants located along a forest road in deciduous broad‐leaved forest. The distance between the populations ranged from 0.4 to 52.4 km. We conducted the following measurements during the flowering peak of each population.

### Floral size measurement

**Fig. 4:**
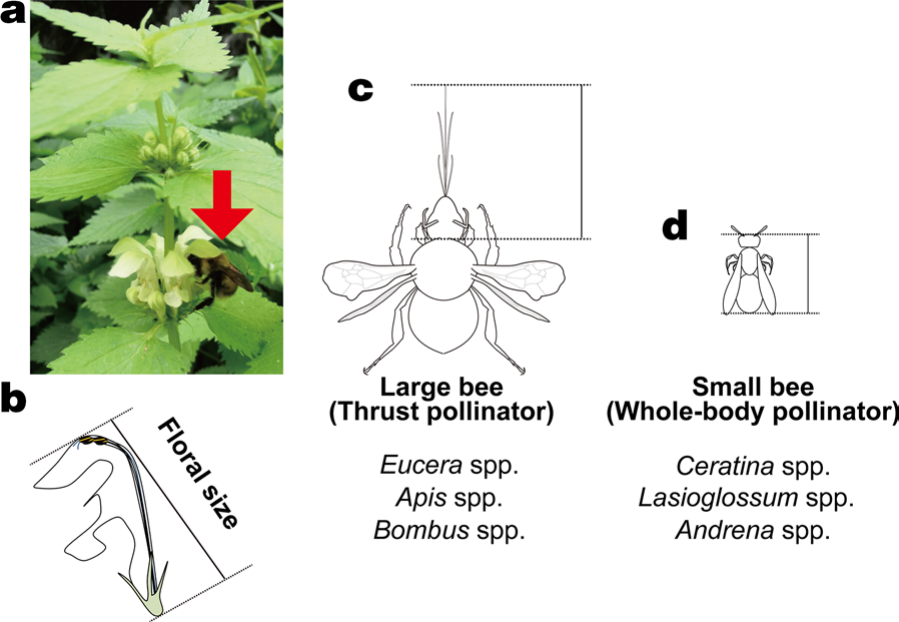
*Lamium album* var.* barbatum* flowers and pollinators. **a** A *Bombus consobrinus* queen (red arrow) visiting a *L. album* var. *barbatum* flower in the Mitsumata population. **b** Measurement of floral size. **c** Mouthpart measurement in large bees that forage for nectar by thrusting their head into the flowers. **d** Measurement of body size of small bees that forage for nectar by crawling into the flowers

First, 18–170 individuals from each population were haphazardly selected and marked with color tape. Then, following the method of Hattori et al. (2015) [41], we measured the floral size of 1–6 flowers per individual plant with a digital caliper (precision, 0.01 mm). The floral size was defined as the distance from the flower's base at the stem to its tip (Fig. 4). Preliminary measurements showed that the variation of floral size among flowers on an individual plant was less than the variation among plants. Therefore, we used the average value of the measured floral sizes of 1–6 flowers on an individual plant as the floral size of that plant. We also measured plant height, as a proxy for plant resource status, of 20 haphazardly selected individuals in each population. Average floral sizes were compared between populations by using Tukey's honestly significant difference (HSD) test. In addition, we used the Moran's I test for spatial autocorrelation to determine to what degree correlations could be explained by the sampling of populations in close proximity to one another. For this test, we used the moran.test function in the "spdep" package in the R software environment ver. 4.0.2 [49].

### Pollinator assemblages and size variation

To observe the pollinator assemblages of *L. album* var. *barbatum*, we selected the largest patch of plants (ranging in area from about 10 to 200 m^2^) in each of the 12 populations and haphazardly established a 1 m × 1 m quadrat (about 100 individuals) within the patch on each census day (Table 1). We then recorded the insects that visited the flowers in this quadrat. Observations were made on several days between 8:00 and 14:00 local time, when flower visitors were active in each population. At each location, we observed all flower visitors for a total of 90–660 min spread over 1–4 days during the peak flowering period. Since bumblebee species (*Bombus* spp.) can be easily distinguished while they are visiting a flower, the species of each bumblebee was recorded as they visited a flower, and the observed species were recorded. In contrast, it is difficult to distinguish among *Eucera* spp. and species of small bees during their flower visits, so we estimated the species-level pollinator assemblage of these taxa from capture survey results (see below).

To define the size of each pollinator species, we measured morphological traits of each species relevant to the pollinating behavior of that species. For this survey, flower-visiting insects were haphazardly captured following their flower visitation, and the size of each of the selected traits was measured with a digital caliper (precision, 0.01 mm). *Bombus* spp., *Eucera* spp., and *Apis cerana japonica* (hereafter, "large bees") are "thrust pollinators"; they forage for nectar by thrusting their heads into flowers and extending their tongues. Thus, we defined the pollinator size of large bees as the sum of the tongue length and the head length. (Fig. 4). In contrast, *Ceratina* spp., *Lasioglossum* spp., and *Andrena* spp. (hereafter, "small bees") are "whole-body pollinators"; they forage for nectar by crawling into the corolla tube. The small bees first land at the entrance to the flowers (upper or lower lip), and then crawl into the flowers to forage, moving through the anthers and stigma to the nectary. As a result, pollen grains become attached to both the head and the ventral side of the abdomen of small bees; thus, we defined the pollinator size of small bees as the body length from the tip of its tongue to the caudal end of the abdomen (Fig. 4). Nectar robbers (*Apis mellifera*, *Bombus hypocrita*, *Xylocopa appendiculata circumvolans*) and small bees on which we did not observed attached pollen grains (*Euodynerus nipanicus*, *Lasioglossum nipponense*, *L. occidens, Nomada comparata* at Onosawa, *Nomada* spp. at Onosawa) were excluded from this calculation of average pollinator size. We checked for attached pollen grains soon after a bee's visit to a flower and identified *L. album* var. *barbatum* pollen grains under a microscope (× 2–10). The bees were observed in a motionless state after anesthesia. Pollen grains were observed by visual inspection, and the pollen grains of *L. album* var. *barbatum* had a very distinct color against the body color of the bees. Pollinator size was measured separately for each plant population, even for insects of the same species. Although *B. diversus* workers were observed in the quadrat surveys at Onosawa and Norikura, and *B. honshuensis* workers at Ohmizusawa, they were not captured and their sizes in those populations were not measured. Therefore, the mean size of all *B. diversus* (*B. honshuensis*) individuals captured from the other populations was used as the size of *B. diversus* at Onosawa and Norikura (*B. honshuensis* at Ohmizusawa).

As the average pollinator size for each plant population, the weighted arithmetic mean was calculated from the relative abundance of each pollinator species in the pollinator assemblage and the size of that species:

$$\mathrm{Average pollinator size}= \sum_{i=1}^{n}Pi(Ni/Nt)$$

where *n* = the total number of insect species visiting a *L. album* var. *barbatum* population (patch), *Pi* = mean size of the *i*th insect species, *Ni* = the number of flowers in the patch that the *i*th insect species visited, and *Nt* = the number of flowers in the patch that any of the insect species visited. Thus, *Ni*/*Nt* is the relative abundance of the *i*th insect species visiting the population. For each population, average pollinator size was calculated for three groups of flower visitors: all flower visitors, only large bees, and only small bees.

### Factors influencing local floral size

To examine factors influencing floral size, we used a linear mixed model (LMM) with a Gaussian error distribution and identity as the link function. Before this analysis, we tested the effect of the variables by likelihood ratio tests. First, we prepared a model with all variables as follows: floral size of each individuals was the response variable, and the average pollinator size (all pollinators), average pollinator size (only large bees), average pollinator size (only small bees), average plant height of each population, and the altitude of each population were predictive variables. We treated the altitude as a proxy for clinal abiotic environmental changes (e.g. meteorological changes). In addition, we treated plant individual and sampling data (year and month) as random effects. The variance inflation factor (VIF) statistic was used to confirm the correlation among predictive variables with VIF = 0.5 as a threshold value [50]. No VIFs above the threshold were detected. A likelihood ratio test using the parametric bootstrap method [51] was performed for models that included all variables and models that lacked one of each predictive variable and random effect. Variables were selected from the difference in deviance between the models obtained by 1000 bootstrap calculations. As a likelihood ratio test results, the average pollinator size (all pollinators), average pollinator size (only large bees) and altitude remained as predictive variables.

The LMM analysis was performed with the lmer function in the "lme4" package in the R software environment ver. 4.0.2 [49]. We further conducted a model selection approach based on AIC. First, we performed model selection on the entire dataset using brute force approach (trying every possible model), starting from a global model including all remained predictive variable by likelihood ratio test, and plant individual and sampling data (year and month) as random effects. These are the explanatory variables that were judged to be valid in the likelihood ratio test results. We then compared the global model with all simpler models based on AIC (i.e. comparing all the combinations of explanatory variables) using the dredge function in the "MuMIn" package in the R software environment ver. 4.0.2 [49]. This function returned the model with the lowest Akaike information criterion (AIC), and we adopted this model (Additional file 2: Table S2). The results of this model selection procedure informed which average pollinator size variable (all pollinators or only large bees) was used in a least-squares regression analysis. Using these results, therefore, we explored covariation between corolla tube length and the average pollinator size of only large bees across populations by a least-squares regression analysis.

### Genetic similarities of *Lamium album* var. *barbatum* populations

To examine the genetic structure of *L. album* var. *barbatum*, we used 10 polymorphic microsatellite primers originally developed for *L. album* [52] (Additional file 4: Table S3). For this analysis, fresh leaf material was collected randomly from 8–16 individual plants in each of the 12 *L. album* var. *barbatum* populations during 2018–2019. DNA was extracted by the CTAB method [53], and the extracted DNA was diluted or concentrated to a final concentration of 10 μg/ml.

Each of the forward microsatellite primers was synthesized after adding one of four different universal fluorescent sequences: 5′-GCCTCCCTCGCGCCA-3′, 5′-GCCTTGCCAGCCCGC-3′, 5′-CAGGACCAGGCTACCGTG-3′, or 5′-CGGAGAGCCGAGAGGTG-3′ [54]. Polymerase chain reaction (PCR) analyses were performed in a thermal cycler using a reaction mixture consisting of 1 μl template DNA, 3 μl of 2 × Type-it Microsatellite PCR Kit (QIAGEN, Valencia, California, USA), 0.7 μl of 0.1 μM forward primer, 0.7 μl of 0.2 μM reverse primer, and 0.7 μl of 0.1 μM fluorescent-labeled universal primer. The DNA amplification program consisted of an initial denaturation step of 5 min at 95 °C, followed by 35 cycles at 95 °C for 30 s, 60 °C for 90 s, and 72 °C for 30 s, and final elongation at 60 °C for 30 min. The PCR products were detected by using an ABI Prism 3130 Genetic Analyzer (Applied Biosystems, Waltham, Massachusetts, USA) and GeneScan™ 500 LIZ™ dye Size Standard (Applied Biosystems). Fragment lengths were calculated with GeneMapper version 4.0 software (Applied Biosystems).

We tested two analysis of molecular variance (AMOVA) models estimating the percentage of molecular variance accounted for by each level of the nested sampling hierarchy. First model, 12 populations were divided according to the two mountain areas (east or west areas). Second model, 12 populations were divided the six floral size groups. Floral size groups were constructed based on the results of Tuley's HSD comparison of the average floral size among the populations. Floral size groups were divided into six groups with significantly different flower sizes (see Table 1, alphabet a, b, c, d, e, fg). AMOVA was run using Arlequin ver 3.5.2.2 [55]. The significance of variance components in the AMOVA models was tested by 1000 random permutations.

In addition, a Bayesian clustering analysis of the fragment length datasets was performed with STRUCTURE software version 2.3.4 [56, 57]. We used this analysis to determine the genetic cluster to which each individual is assigned. Simulations were conducted with 100 k burn-in iterations and 100 k Markov chain Monte Carlo repetitions. The number of genetic clusters (*K*) was calculated 10 times for each of 1–12, and the Δ*K* value [58] was used as the criterion for selecting the appropriate number of clusters, that is, the number of genetic clusters from which the 12 populations of *L. album* var. *barbatum* were derived.

## Supplementary Information

**Additional file 1: Table S1.** Sizes of the captured flower visitors (pollinators) in each population (mean ± SE). Insects using the "whole-body" visitation mode are small bees, and those using the "thrust" visitation mode are large bees (See Table 1). Castes of *Bombus* spp. are indicated by W, worker, or Q, queen. An asterisk following the species name indicates that no pollen grains were found on the bodies of insects of that species.

**Additional file 2: Table S2.** Results of the LMM model selection using the dredge function in the "MuMIn" package.

**Additional file 3: Figure S1.** Geographic genetic structure on study sites. Pie chart indicate that the proportion of the two clusters identified by STRUCTURE as averaged for each population. The west area comprises populations in the Mt. Norikura region, and the east area comprises populations in the Utsukushigahara highland region.

**Additional file 4: Table S3.** Information on the 10 microsatellite markers used in this study. These markers were developed by Horsley (2013) [52].

## Data Availability

The datasets used and/or analysed during the current study available from the corresponding author on reasonable request.
