# Supplementary material for: Intraspecific convergence of floral size correlates with pollinator size on different mountains: a case study of a bumblebee-pollinated Lamium (Lamiaceae) flowers in Japan
Source: BMC Ecol Evol. 2021 Apr 24;21:64. doi: 10.1186/s12862-021-01796-8 (PMC8067403; doi:10.1186/s12862-021-01796-8)
Supplement: Supplementary file 3 — Additional file 3: Figure S1. Geographic genetic structure on study sites. Pie chart indicate that the proportion of the two clusters identified by STRUCTURE as averaged for each population. The west area comprises populations in the Mt. Norikura region, and the east area comprises populations in the Utsukushigahara highland region. [file 12862_2021_1796_MOESM3_ESM.docx]

**Additional file 3**


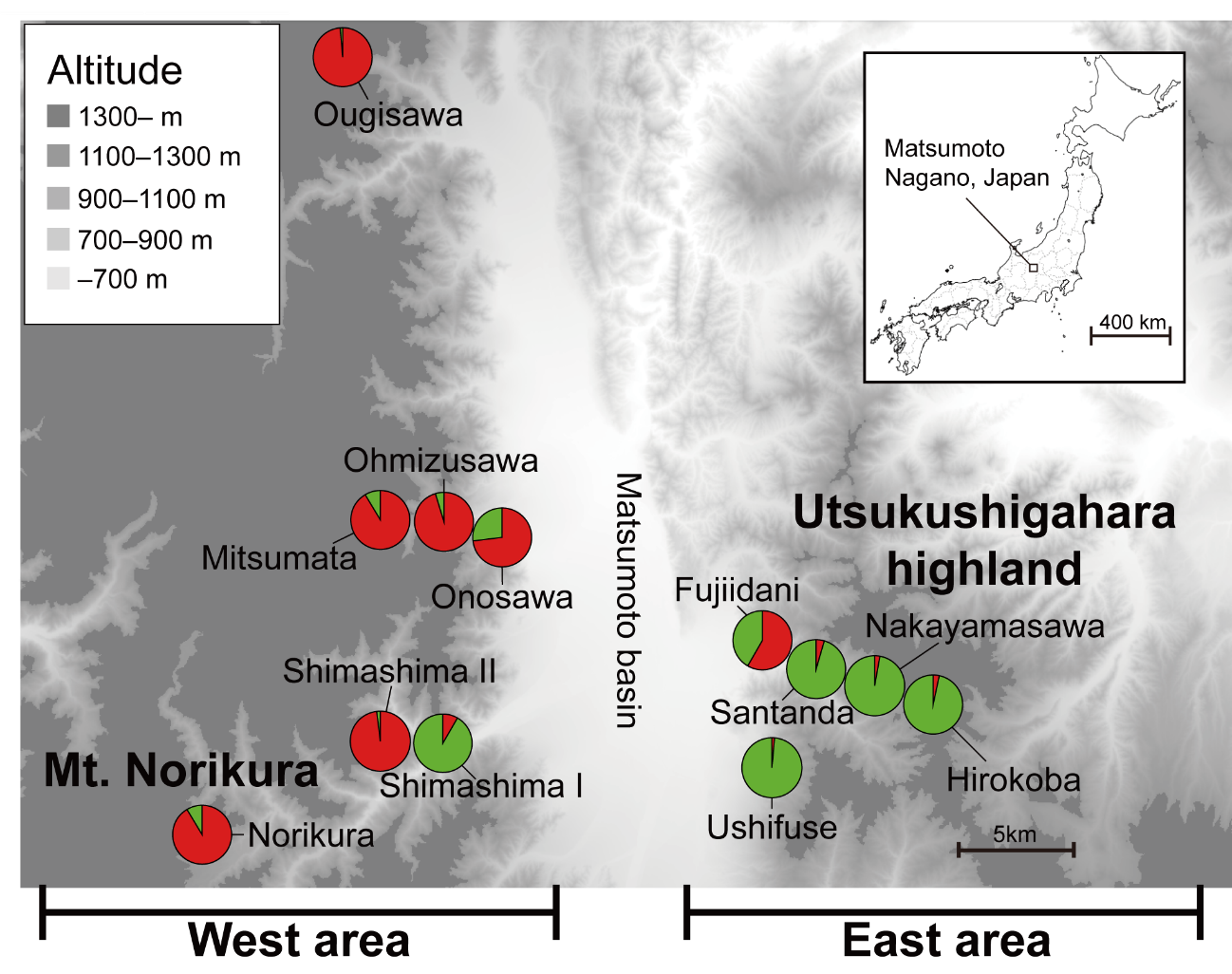


**Figure S1: Geographic genetic structure on study sites.** Pie chart indicate that the proportion of the two clusters identified by STRUCTURE as averaged for each population. The west area comprises populations in the Mt. Norikura region, and the east area comprises populations in the Utsukushigahara highland region.
